# A Prediction Model for Detecting Developmental Disabilities in Preschool-Age Children Through Digital Biomarker-Driven Deep Learning in Serious Games: Development Study

**DOI:** 10.2196/23130

**Published:** 2021-06-04

**Authors:** Ho Heon Kim, Jae Il An, Yu Rang Park

**Affiliations:** 1 Department of Biomedical Systems Informatics Yonsei University College of Medicine Seoul Republic of Korea

**Keywords:** developmental delay, diagnosis prediction, deep learning, serious games, digital health, digital phenotyping, digital biomarkers

## Abstract

**Background:**

Early detection of developmental disabilities in children is essential because early intervention can improve the prognosis of children. Meanwhile, a growing body of evidence has indicated a relationship between developmental disability and motor skill, and thus, motor skill is considered in the early diagnosis of developmental disability. However, there are challenges to assessing motor skill in the diagnosis of developmental disorder, such as a lack of specialists and time constraints, and thus it is commonly conducted through informal questions or surveys to parents.

**Objective:**

This study sought to evaluate the possibility of using drag-and-drop data as a digital biomarker and to develop a classification model based on drag-and-drop data with which to classify children with developmental disabilities.

**Methods:**

We collected drag-and-drop data from children with typical development and developmental disabilities from May 1, 2018, to May 1, 2020, via a mobile application (DoBrain). We used touch coordinates and extracted kinetic variables from these coordinates. A deep learning algorithm was developed to predict potential development disabilities in children. For interpretability of the model results, we identified which coordinates contributed to the classification results by applying gradient-weighted class activation mapping.

**Results:**

Of the 370 children in the study, 223 had typical development, and 147 had developmental disabilities. In all games, the number of changes in the acceleration sign based on the direction of progress both in the x- and y-axes showed significant differences between the 2 groups (*P*<.001; effect size >0.5). The deep learning convolutional neural network model showed that drag-and-drop data can help diagnose developmental disabilities, with an area under the receiving operating characteristics curve of 0.817. A gradient class activation map, which can interpret the results of a deep learning model, was visualized with the game results for specific children.

**Conclusions:**

Through the results of the deep learning model, we confirmed that drag-and-drop data can be a new digital biomarker for the diagnosis of developmental disabilities.

## Introduction

Developmental disabilities are a set of common heterogeneous disorders developing in 10%-15% of preschool-age children and characterized by difficulties in one or more domains, including learning, behavior, and self-care [1-3]. The prevalence trend of all developmental disabilities increased from 1997 to 2017 in the United States, and the trend in low- and middle-income countries has also increased in the number of children surviving high-risk neonatal conditions from improved obstetric and neonatal care [4,5]. Although the etiology and cause of developmental disabilities are complicated and not well understood, early intervention is conventionally considered as an effective clinical treatment [6,7]. Early detection of developmental disabilities is key because early intervention can improve a child’s prognosis due to rapid brain growth and neuroplasticity [8-10]. However, early detection or screening has multiple challenges, including time constraints, financial burden, scarcity of human resources, lack of consensus on the tools for the general childhood population, and diagnostic stability [11,12]. Given the phenotypical nature of developmental disabilities, the assessment processes show high variability [13,14]. Neuropsychological tests are often difficult and tedious for preschool-age children to complete, leading to inaccurate assessment [14]. Moreover, although it is important to perform continuous clinical examinations and comprehensive tracking for more accurate assessment [15-17], poor follow-up adherence rates have been reported. This low follow-up rate can induce a loss of chance for early intervention [18].

Meanwhile, a growing body of evidence has indicated a relationship between developmental disability and motor control, because the cerebellum is closely related to higher cognitive function [19]. Motor skill is considered to be a factor in the early diagnosis of developmental disability [20,21]. Despite this evidence, the measurement of motor skill requires expensive laboratory resources or clinical expertise and is not easily applicable in repeated measurements. As an alternative to measuring motor skill and without the constraints of time and place, a serious game that is able to capture upper extremity movements while touching a display could help in detecting children with developmental disabilities.

Therefore, this study aimed to identify the possibility of drag-and-drop data as a digital biomarker and to develop a classification model based on drag-and-drop data with which to classify children with developmental disabilities.

## Methods

### Serious Games

This study included children who had experiences with a serious game known as DoBrain (DoBrain Inc). DoBrain is a mobile-based game that provides programs for the cognitive development of children. The games of this application consist of chapters of 7 to 8 subgames targeting spatial awareness, perceptual speed, repair, creativity, reasoning, composition, memory, and visual discrimination of the cognitive area. Each subgame can be classified into a tapping game where users have to solve a problem by touching objects to answer a question or a drag-and-drop game where users have to drag and drop cartoon objects with their fingers. In addition, the difficulty of the game is divided into 3 levels (A, B, and C) depending on the cognitive level of the user. Chapter 1 comprises 7 subgames, including 4 tapping games or non–drag-and-drop games (first, fourth, fifth, and seventh) and 3 drag-and-drop games (second, third, and sixth). The second game is an imitation game in which users must infer the correct answer from similar images of an object and is designed to improve logical reasoning. The third subgame requires the user to infer the correct answer from remnant images and is designed to improve memory function. The sixth subgame is designed to improve spatial awareness by requiring the user to locate the object on the target region (Multimedia Appendix 1).

### Study Design

**Figure 1:**
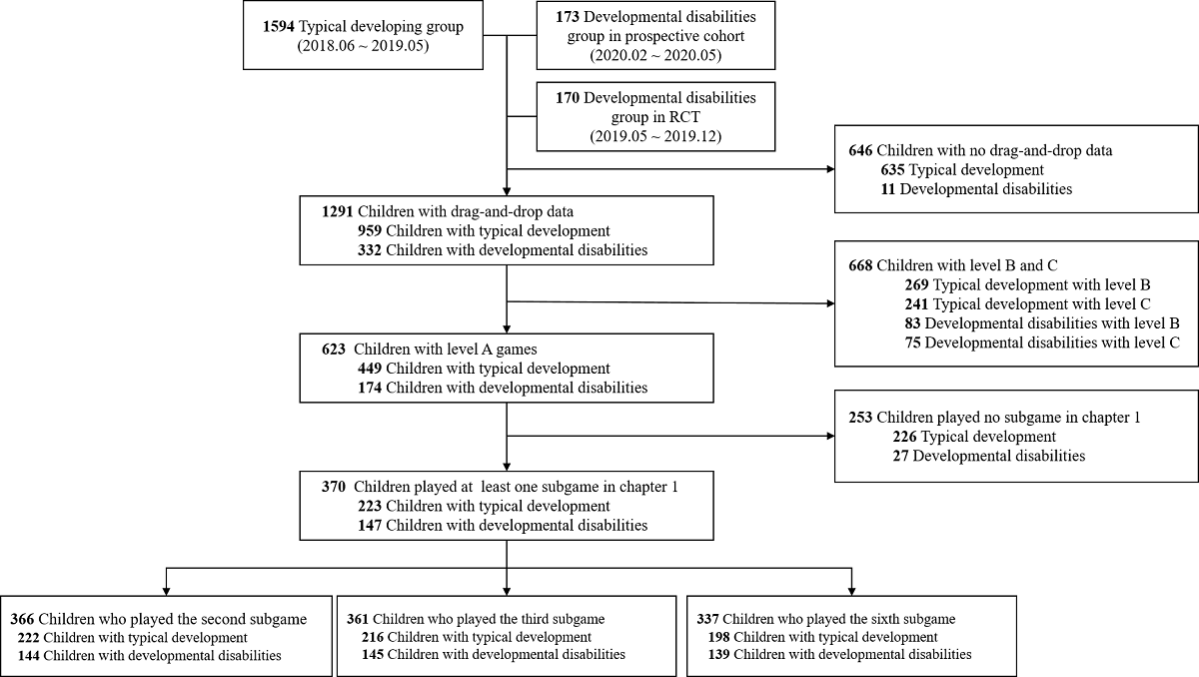
Eligible user selection flow.

In this retrospective study, we obtained deidentified participant data from 3 studies: (1) a retrospective study conducted between June 1, 2018, and June 1, 2020, with children having profile information in the application; (2) randomized clinical trials conducted from March 1, 2019, to December 30, 2019, for evaluation of cognitive improvement in children with developmental disabilities; and (3) a prospective study conducted from February 1, 2020, on a development classification model. The profiles of children with typical development included in the first study were entered by their parents, and the children included in the second and third studies were diagnosed by pediatric psychologists. In each study, 1594 children with typical development and 343 children with developmental disabilities (173 and 170 children, respectively) were included. Among the 1937 children with valid profiles, we also excluded children without drag-and-drop data due to server instability or unexpected shutdown of the game (n=646). Moreover, in 1291 children with drag-and-drop data, we included only those children who played games at difficulty level A to make experienced games homogenous because games at levels B and C have more objects to drag and drop than do games at level A (n=623). Finally, we included children who played at least one subgame among the second, third, and sixth subgames because the other subgames in chapter 1 are played with tapping answer objects (n=370). In these drag-and-drop subgames, we analyzed the drag-and-drop log data to classify those children with typical development and those with disabilities (Figure 1)**.**

### Ethics Statement

The retrospective study was approved by the institutional review board of Yonsei University College of Medicine in South Korea (no. Y-2020-0076). The Division of Biomedical System Informatics (Department of Medicine of Yonsei University) and DoBrain work together as a nonprofit, joint research group for the early detection of disabilities in children and improved cognitive function. To conduct this research, we obtained deidentified data from DoBrain, and we have no conflicts of interest related to our dealings with DoBrain Inc.

### Analytical Procedure

In our study, we compared the baseline characteristics of children with and without developmental disabilities using the *t* test or Mann-Whitney test for continuous variables (eg, age and device size) and used chi-square test for categorical variables (eg, sex). Furthermore, we derived features related to drag-and-drop data to capture children’s kinetics (Multimedia Appendix 2). We additionally analyzed these derived data to identify differences in features from finger strokes between the 2 groups. Before comparative tests, we explored the normality of distribution by visual methods and statistical tests (Kolmogorov-Smirnov test) [22]. We determined normality with consideration to the shape of the histogram and the results of statistical tests [23]. In addition, we conducted an *F* test for homoscedasticity (equal variance in 2 populations). We then conducted a *t* test for normally distributed data and a Mann-Whitney test for nonnormally distributed data.

*P* values <.05 were considered statistically significant for 2-sided hypothetical tests. In addition, we calculated effect sizes to determine the possibility of type I statistical error. Cohen *d* for continuous variables for normally distributed data and for categorical variables were considered small depending on the type of effect size (η^2^≈0.01; –0.20 < Cohen *d* < 0.20) [24]. For nonparametric comparative methods, such as the Mann-Whitney test, the common language effect size (CLES) was calculated to identify the probability that a score sampled at random from one distribution would be greater than a score sampled from another distribution. CLES reflects the chance that a value for a randomly selected child with typical development would be higher than that from one with developmental disabilities [25].

For detecting children with developmental disabilities, we developed a deep learning classification model based on a 1D convolutional neural network for drag data. Using drag data, we tried to leverage multiple inputs (time variant variables: touch coordinates and their derived variables; time-fixed variables: statistics acquired at the end of the game, such as total touch area or demographic data) by joint fusion. We subsequently modeled the classification algorithm using deep learning and not conventional machine learning. In addition, we applied a strategy to decompose coordinates (fine motor movement) along the x- and y-axes. Through this decomposition of coordinates, we were able to leverage information along each axis by creating derived variables, such as velocity and acceleration, along the axes and volatility of sign change. Because this approach did not use positional information (contextual information) in 2D, we developed a model with a 1D convolutional neural network.

Drag-and-drop data, including all touch traces, were leveraged in our model. The traces of individual touch attempts were captured and stored in the forms of logs when the user touched an object on the display. We tried to use drag-and-drop data regardless of intention to touch. Because the direction is guided through sounds at the beginning of the game, we built the model to capture unnecessary touches. Therefore, we used all touch records to classify children with or without developmental disabilities.

**Figure 2:**
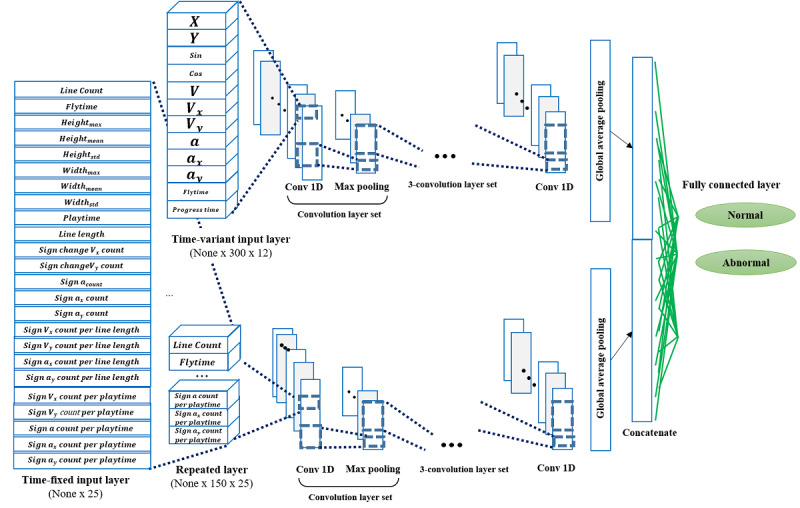
Deep learning model architecture. conv: convolution.

At the end of the model, multiple inputs were concatenated by the individual user ID, and the output node calculated the binary prediction of developmental disabilities using a fully connected layer. For time-variant variables, derived variables calculated from each drag-and-drop feature were input. In contrast, for the time-fixed variable, demographic characteristics and game results that were generated after the end of the subgame were input (Figure 2).

For hyperparameter optimization, we searched hyperparameters with a grid search. A detailed description of this search is included in Multimedia Appendix 3. Deep learning models based on each subgame were also evaluated using 10-fold cross-validation. The area under the receiver operating characteristics curve (AUROC), area under the precision-recall curve, *F* score, precision, recall (sensitivity), and specificity for each subgame were calculated as the aggregation of the 10-fold cross-validation results.

Finally, we not only focused on building a classification model for developmental delay but also developed an interface with which to examine the network’s decision to assess the fine motor movement feature detected by the network. We applied the gradient-weighted class activation mapping (Grad-CAM) method to determine which coordinates of fine motor movement touch were useful for predicting developmental disabilities in children [26]. For this, we overlaid coordinates with an attention map from the Grad-CAM, which showed the coordinates of positive correlates with the output of the network. All statistical analyses and model development were conducted using Python 3.6.8 and TensorFlow 1.14.0.

### Ethics Statements

Our relationship with DoBrain Inc. involves no conflicts of interest. We and DoBrain Inc. have experience in conducting national research projects together (“Cognitive learning service for children with developmental disabilities using on AI” [unpublished data], 2019-2020) with funding from the National Information Society Agency, South Korea. Within these national projects, we obtained the deidentified data from DoBrain Inc.

## Results

### Baseline Characteristics

A total of 368 eligible children were included in the study. Of these, 366, 361, and 337 played the second, third, and sixth subgames, respectively (overall, 223 children had typical development, and 147 had developmental disabilities). There was a statistical difference in the chronological age of the 2 groups (*P*<.001; CLES=0.839) and in the mean playtime (*P*<.001; CLES=0.687). In each played subgame, a difference in the ratio of children who played games was only observed for the sixth subgame (*P*=.04; CLES=0.002; Table 1).

**Table 1:** Demographic characteristics in eligible users.

Variables		Children with typical development (n=223)	Children with developmental disabilities (n=147)	Total (n=370)	*P* value	Effect size
Age (months), median (IQR)		40.0 (12.0)	72.0 (32.5)	45.0 (26.5)	<.001	0.839^a^
**Diagnosis, n (%)**						
	Intellectual disability	0 (0.0)	44 (0.33)	44 (0.11)	N/A^b^	N/A
	Autism spectrum disorder	0 (0.0)	41 (0.27)	41 (0.11)	N/A	N/A
	Developmental disorder	0 (0.0)	33 (0.22)	33 (0.09)	N/A	N/A
	Brain lesions	0 (0.0)	25 (0.17)	25 (0.09)	N/A	N/A
	Monogenic disorder	0 (0.0)	4 (0.02)	4 (0.01)	N/A	N/A
**Children playing subgame, n (%)**						
	Second subgame	222 (99.55)	144 (97.96)	366 (98.92)	.03	<0.001
	Third subgame	216 (97.31)	145 (98.64)	361 (97.84)	.24	<0.001
	Sixth subgame	198 (88.79)	139 (95.56)	337 (91.98)	.04	0.002
Device size (inches), median (IQR)		6.1 (4.2)	6.1 (4.2)	6.1 (4.2)	<.001	0.469^a^
Game playtime, (s/per game), median (IQR)		11.8 (8.5)	8.1 (6.5)	10.3 (8.2)	<.001	0.687^a^
Games played, median (IQR)		9.0 (9.0)	1.0 (14.2)	7.0 (12.5)	<.001	0.682^a^

^a^Common language effect size of continuous variables; η^2^ for effect size of categorical variables.

^b^N/A: not applicable.

### Characteristics of Drag-and-Drop Game Play

Although playtime did not consistently show statistically significant differences in each subgame, the playtime in children with typical development was significantly longer than that of children with disabilities for the sixth subgame (*P*<.001; CLES=0.616; Table 2). In the touch region, the variables related to measurements of the touch area did not show a statistically significant difference in median values. However, in the sixth subgame, the median max of height and width that children used for playing games showed a significant difference (*P*<.001; CLES>0.65). In addition, although the median change of velocity sign change along the x-axis did not show a difference (*P*=.40, *P*=0.17, and *P*=0.08, respectively) in all subgames, the IQR of children with typical development was smaller than the that of children with developmental disabilities (Multimedia Appendix 4). In 4 accelerator variables, including sign change of acceleration along the x-axis and y-axis, with both count and per line count, the number of sign changes for children with typical development was larger than that for children with developmental disabilities.

**Table 2:** Comparison of movement features between children with typical development and children with developmental disabilities in each game.

Characteristic			Children with typical development		Children with developmental disabilities		*P* value		CLES^a^
**Second game**									
	Distribution, n		222	144		N/A^b^		N/A	
	**Play information, median (IQR)**								
		Playtime (second/game)	2.03 (6.39)	1.98 (5.11)		.33		0.513	
		Line number (n/game)	1.0 (2.0)	2.0 (2.0)		.24		0.376	
		Line length (n/game)	3.04 (3.17)	3.1 (4.24)		.28		0.518	
		Release to touch time (sec)	0.0 (3.31)	0.52 (4.46)		.15		0.409	
	**Touch region, median (IQR)**								
		Height mean, ratio (%)	0.38 (0.1)	0.38 (0.08)		.26		0.540	
		Height max, ratio (%)	0.58 (0.08)	0.57 (0.06)		.10		0.520	
		Width max, ratio (%))	0.74 (0.08)	0.72 (0.08)		.13		0.534	
		Height mean, ratio (%)	0.38 (0.1)	0.38 (0.08)		.006		0.540	
**Third game**									
	Distribution, n		216	145		N/A		N/A	
	**Play information, median (IQR)**								
		Playtime (second/game)	2.75 (6.63)	2.44 (5.99)		.22		0.524	
		Line number (n/game)	1.0 (2.0)	1.0 (2.0)		.46		0.344	
		Line length (n/game)	3.12 (4.23)	3.62 (6.28)		.04		0.553	
		Release to touch time (sec)	0.0 (2.84)	0.0 (3.11)		.45		0.372	
	**Touch region, median (IQR)**								
		Height mean, ratio (%)	0.29 (0.09)	0.27 (0.06)		.02		0.520	
		Height max, ratio (%)	0.35 (0.23)	0.35 (0.17)		.26		0.565	
		Width max, ratio (%))	0.72 (0.1)	0.71 (0.08)		.18		0.528	
		Height mean, ratio (%)	0.29 (0.09)	0.27 (0.06)		.04		0.520	
**Sixth game**									
	Distribution, n		198	139		N/A		N/A	
	**Play information, median (IQR)**								
		Playtime (second/game)	7.85 (15.25)	4.33 (10.03)		<.001		0.616	
		Line number (n/game)	3.0 (3.0)	2.0 (3.0)		.001		0.514	
		Line length (n/game)	4.64 (8.5)	4.5 (7.66)		.41		0.507	
		Release to touch time (sec)	2.9 (8.33)	1.4 (6.27)		.001		0.549	
	**Touch region, median (IQR)**								
		Height mean, ratio (%)	0.43 (0.06)	0.34 (0.16)		<.001		0.695	
		Height max, ratio (%)	0.53 (0.09)	0.49 (0.2)		<.001		0.766	
		Width max, ratio (%))	0.78 (0.1)	0.73 (0.09)		<.001		0.658	
		Height mean, ratio (%)	0.43 (0.06)	0.34 (0.16)		<.001		0.695	

^a^CLES: common language effect size.

^b^N/A: not applicable.

### Model Performance

**Figure 3:**
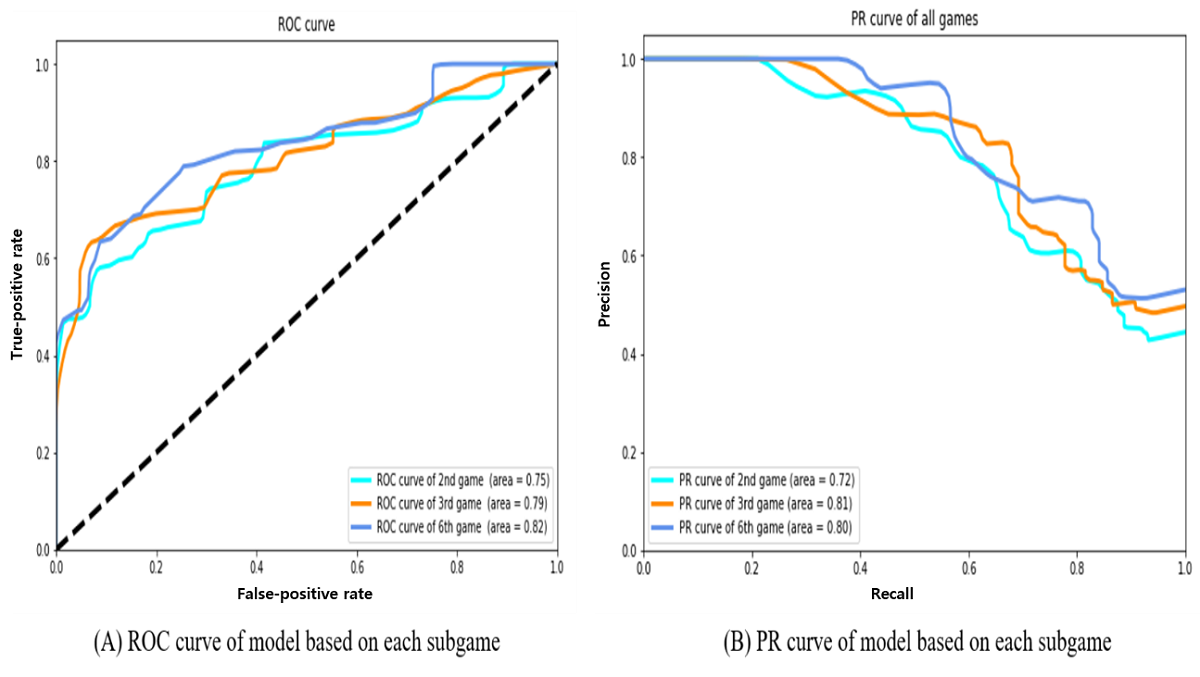
ROC curves and PR curves of the deep learning model. PR: precision-recall. ROC: receiver operating characteristic.

Overall, the average AUROCs for the second, third, and sixth games were calculated as 0.746 (σ=0.116), 0.793 (σ=0.117), and 0.817 (σ=0.070), respectively, in a 10-fold cross-validation; meanwhile, average *F* scores of the deep learning models for each targeted subgame were calculated as 0.627, 0.675, and 0.708, respectively (Table 3). The model for the sixth subgame showed relatively high performance compared to other models using the second or third subgames in terms of average model performance metrics (AUROC, accuracy, *F* score, precision, recall). Recall (also called sensitivity), which refers to the probability of a positive test given that the patient has a disease, was highest in the model for the sixth game (0.757; σ=0.123) (Figure 3). Specificity, which refers to the probability of a negative test given that the patient is normal, was relatively better in the model for the third subgame (0.783; σ=0.069) than in the other models using the second or sixth subgame (second subgame: 0.755, σ=0.089; sixth subgame: 0.740, σ=0.099).

**Table 3:** Average model performance result by 10-fold cross-validation in each drag-and-drop subgame.

Deep learning model for individual subgame	Performance, mean (SD)					
	AUROC^a^	Accuracy	*F* score	Precision	Recall	Specificity
Second subgame	0.746 (0.116)	0.719 (0.094)	0.627 (0.165)	0.616 (0.136)	0.683 (0.151)	0.755 (0.089)
Third subgame	0.793 (0.117)	0.747 (0.082)	0.675 (0.126)	0.686 (0.142)	0.688 (0.167)	0.783 (0.069)
Sixth subgame	0.817 (0.070)	0.769 (0.078)	0.708 (0.153)	0.675 (0.183)	0.757 (0.123)	0.740 (0.099)

^a^AUROC: area under the receiver operating characteristics curve.

### Prediction of Developmental Disabilities From Finger Strokes

**Figure 4:**
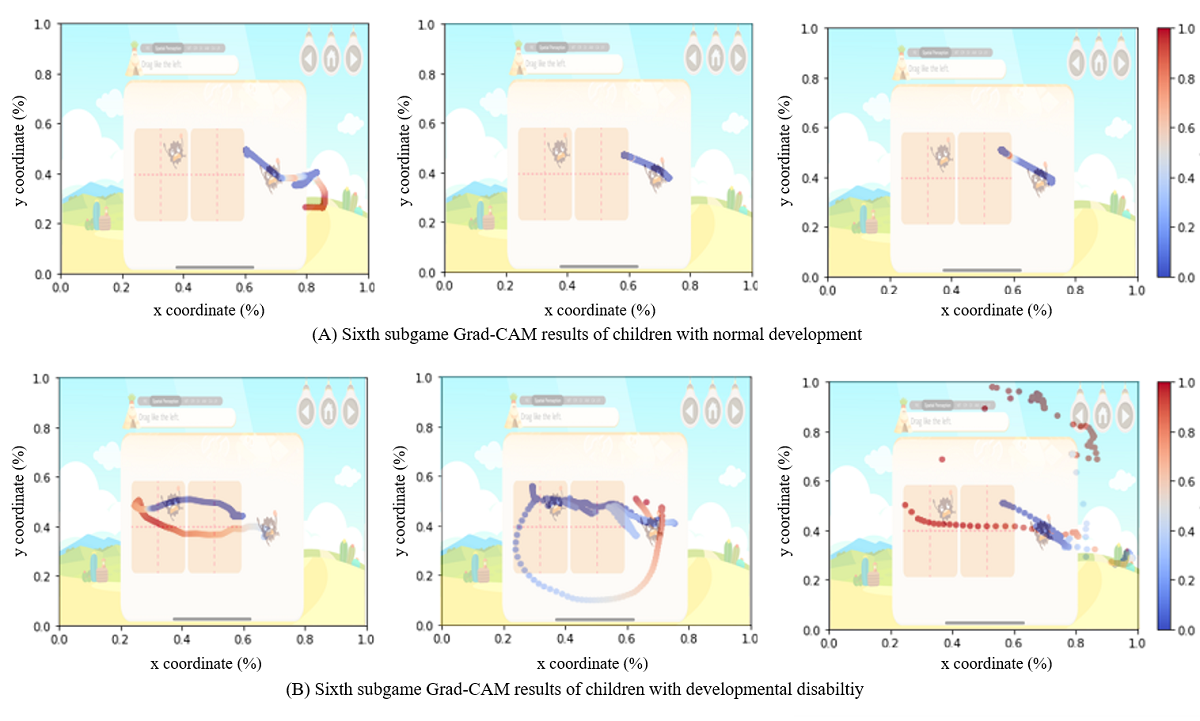
Sixth subgame with Grad-CAM results of children with normal and abnormal development disability. Grad-CAM: gradient-weighted class activation mapping.

The visualization of variable weights for developmental disabilities on game result images relied on a gradient-based projection of the classification scores to the input pixels. Children with typical development correctly implemented the optimal path proximity to answers, as recorded by the game; in Figure 4A, coordinates similar to the optimal path are visualized in blue, as presented in Grad-CAM. In contrast, we could confirm that children with developmental disabilities played games and drew gestures in various locations before drawing the optimal path (Figure 4B). Unlike the coordinates of children with typical development, coordinates located in the nonoptimal path are shown in red, as presented by Grad-CAM.

## Discussion

### Principal Findings

This study showed that fine motor movements captured from touching a mobile display can be a novel digital biomarker for the classification of developmental delay. Our classification model leveraged moment-by-moment drag-and-drop data to capture fine motor movements for serious game play. This suggests that serious games can be used as diagnostic assistant tools or screen tools for detecting developmental disabilities. Moreover, our model can be adaptive to clinicians because it can visualize how much each coordinate of fine motor movement contributes to classification.

### Drag-and-Drop Data as a Digital Biomarker for Detecting Developmental Delay

Early motor delay is often a sign of neurological dysfunction [27]. Fine motor skills are related to the use of upper extremities to engage and manipulate the environment [15,27]. This function is necessary for children to play or accomplish work. At the age of 3 years, children can copy circles and imitate a cross in the course of reaching developmental milestones [28]. Similarly, the games in our study require the user to select an object of the same color or solve the game by imitating a given environment (such as a picture on the left in Multimedia Appendix 1). Although the screening delivery platform has been changed into a digital device, it still seems capable of reflecting developmental milestones.

In the clinic or community, parent- or teacher-reported screening tools are used for large populations, including the Behavior Rating Inventory of Executive Function II (BRIEF-II) and the Denver Developmental Screening Tests II (DDST-II),. These screening tools collect information on the kinesthetic ability of children from parents or teachers. However, as these tools leverage information obtained from parents or teachers to calculate scores reflecting kinesthetic abilities, the results of the screening tools may have inter- or intraobserver variability. [29] Despite this, the use of drag-and-drop data in our model has a strength in that our model presents more objective results.

Previous studies have reported that children with autism show a rate of change in acceleration in movements that is significantly greater than that in children with typical development [30,31]. Although previous studies have reported on acceleration of the device (inertia) captured from a gyroscope in the device or infrared motion tracking system, our study showed higher rates of sign changes in acceleration (in all subgames) and velocity (in the sixth subgame) in finger strokes for dragging and dropping objects in children with typical development compared to children with developmental disabilities. Given that children with developmental disabilities had shorter playtimes and less variability, children with developmental disabilities might not have intuitively understood the problem and indiscriminately ran their finger across the screen. It is also possible that the difficulty of the game may be insufficient to screen problems even in children with developmental disabilities because the purpose of the game is to drag objects in a single straight line.

### Early Detection and Diagnostic Stability

There are multiple challenges to screening children with developmental disabilities in routine clinical practice [2]. In step with the high clinical demand, there are long wait times for children who require examination by specialists. If screening tools are negative for developmental delay and the parents continue to be concerned about their child’s behavior, a more intensive follow-up plan is needed, such as shorter-interval, repetitive screening tests. Other barriers include a lack of consensus on the best screening tools and insufficient physician confidence [2,32,33]. A previous study on diagnostic stabilities in children with and without autism spectrum disorder in the United States showed that 21% of children were initially not diagnosed with autism spectrum disorder [12]. From this point of view, developmental disability classification using mobile devices can be used as an element to potentially overcome these challenges.

Further, for early identification of developmental disability, cross-culturally appropriate and affordable tools are important, although tools satisfying these conditions are limited [34]. Applying tools developed in Western-based norms to other cultural contexts can induce overdetection in children, as there is a disparity in global pediatric mental health, especially in low- and middle-income countries [35]. Considering that many children do not regularly visit medical or mental health professionals in low- and middle-income countries [36], a screening tool that is quick and inexpensive would be desirable. In addition, tools with child-friendly characteristics for children to complete by themselves and under repetitive use with lower resources would be suitable. In this respect, a serious game with drag-and-drop data can be a candidate for tools that satisfy these requirements.

### Limitations

This study has several limitations. First, the diagnostic profile of children with typical development used in our study was based on patient reports. Because children with typical development were not confirmed by physicians, this could have led to bias in the performance or results of the model due to the reliability of the label. However, previous studies included clinical trials using only limited samples, whereas our study analyzed hundreds of children with developmental disabilities diagnosed by a pediatrician. Therefore, compared to previous studies, our study represents an improvement in the robustness of the prediction results. Second, this study excluded children with developmental disabilities who were unable to control the mobile device by themselves. Because data acquisition assumed children could handle the mobile device and understand the instructions to the games, the characteristics of children with severe or moderate developmental disabilities were not considered in this study. Further research needs to be conducted after analyzing children with consideration of the degree of developmental disorders.

### Conclusions

As continuous and comprehensive tracking for more accurate assessment is important in screening developmental disabilities, a screening tool that can be easily, repetitively, and objectively used is needed. To the best of our knowledge, this retrospective study is the first to show that a deep learning-based screening model leveraging digital biomarkers could be feasible for detecting developmental disabilities in children. Therefore, finger strokes on a mobile touch display can be a novel digital biomarker of use in screening for developmental disabilities.

## Appendix

Multimedia Appendix 1 Captured images of DoBrain chapter 1 subgames.

Multimedia Appendix 2 Features used as deep learning inputs and their descriptions.

Multimedia Appendix 3 Velocity and acceleration of movement.

Multimedia Appendix 4 Hyperparameter optimization.

## Abbreviations

AUROC: area under the receiver operating characteristics curve
BRIEF-II: Behavior Rating Inventory of Executive Function II
CLES: common language effect size
DDST-II: Denver Developmental Screening Tests II
Grad-CAM: gradient-weighted class activation mapping
