# Supplementary figures and images for: A Prediction Model for Detecting Developmental Disabilities in Preschool-Age Children Through Digital Biomarker-Driven Deep Learning in Serious Games: Development Study
Source: JMIR Serious Games. 2021 Jun 4;9(2):e23130. doi: 10.2196/23130 (PMC8214184; doi:10.2196/23130)

**Supplement 1.** Captured images of Dobrain chapter 1 subgames


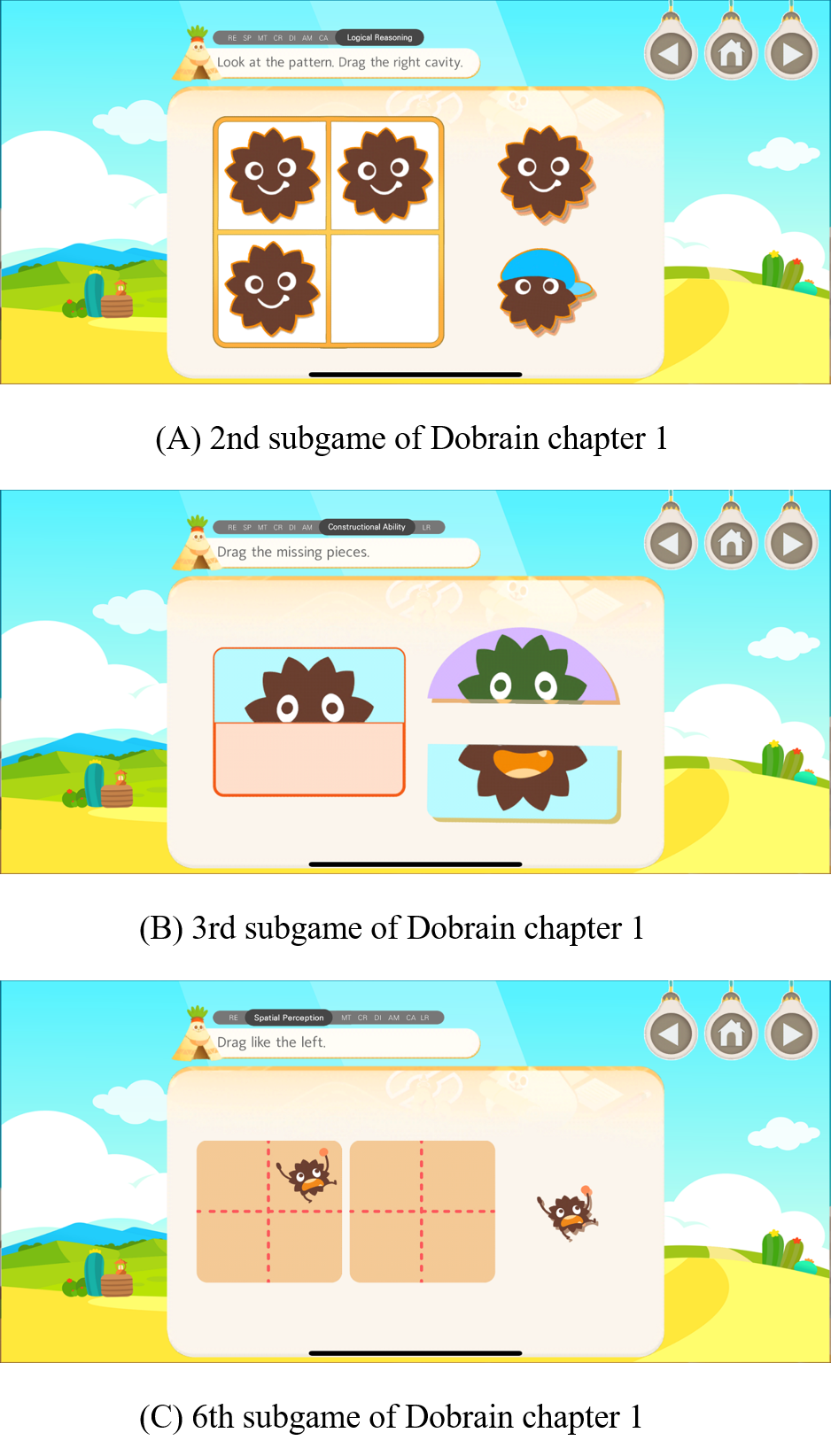

Supplement: Multimedia Appendix 1 [file games_v9i2e23130_app1.docx]
