# Supplementary material for: A Prediction Model for Detecting Developmental Disabilities in Preschool-Age Children Through Digital Biomarker-Driven Deep Learning in Serious Games: Development Study
Source: JMIR Serious Games. 2021 Jun 4;9(2):e23130. doi: 10.2196/23130 (PMC8214184; doi:10.2196/23130)

**Supplement 2.** Features used as deep learning inputs and their descriptions

| Feature name | Description | Time variant variables (T) and Time-fixed variables (F) |
| --- | --- | --- |
| X | X value of coordinates recognized by the sensor | T |
| Y | Y value of coordinates recognized by the sensor | T |
| sin | Sin of the angle between the coordinates and the origin | T |
| cos | Cos of the angle between the coordinates and the origin | T |
| V | Velocity at that coordinate | T |
| V_x | Velocity at that coordinate x-axis(horizontal) value | T |
| V_y | Velocity at that coordinate y-axis(longitudinal) value | T |
| a | Accelerometer at that coordinate | T |
| a_x | Accelerometer at that coordinate x-axis(horizontal) value | T |
| a_y | Accelerometer at that coordinate y-axis(longitudinal) value | T |
| Fly Time | Time taken to recognize the coordinates from the previous coordinates | T |
| Progress_Time | Time taken to recognize the coordinates after the start of the play | T |
| Line  Count | Number of lines used to play the game | F |
| Line  Length | Sum of the length of all lines used to play the game | F |
| Fly Time | Average of Fly Time calculated while playing the game | F |
| Maximum  Height | Maximum value of height(Y) | F |
| Mean  Height | Mean value of height(Y) | F |
| STD  Height | Standard deviation value of height(Y) | F |
| Maximum  Width | Maximum value of width(X) | F |
| Mean  Width | Mean value of width(X) | F |
| STD  Width | Standard deviation value of width(X) | F |
| Playtime | Total time spent playing the game | F |
| Vx_count | Velocity x-axis(horizontal) value sign(+/-) change count | F |
| Vy_count | Velocity y-axis(longitudinal) value sign(+/-) change count | F |
| a_count | Accelerometer direction value sign(+/-) change count | F |
| ax_count | Accelerometer x-axis(horizontal) value sign(+/-) change count | F |
| ay_count | Accelerometer y-axis(longitudinal) value sign(+/-) change count | F |
| Vx_count per line count | Velocity x-axis(horizontal) value sign(+/-) change count per sum of all lines used to play the game | F |
| Vy_count per line count | Velocity y-axis(longitudinal) value sign(+/-) change count per sum of all lines used to play the game | F |
| a_count per line count | Accelerometer direction value sign(+/-) change count per sum of all lines used to play the game | F |
| ax_count per line count | Accelerometer x-axis(horizontal) value sign(+/-) change count per sum of all lines used to play the game | F |
| ay_count per line count | Accelerometer y-axis(longitudinal) value sign(+/-) change count per sum of all lines used to play the game | F |
| Vx_count per play time | Velocity x-axis(horizontal) value sign(+/-) change count per total playing time | F |
| Vy_count per play time | Velocity y-axis(longitudinal) value sign(+/-) change count per total playing time | F |
| a_count per play time | Accelerometer direction value sign(+/-) change count per total playing time | F |
| ax_count per play time | Accelerometer x-axis(horizontal) value sign(+/-) change count per total playing time | F |
| ay_count per play time | Accelerometer y-axis(longitudinal) value sign(+/-) change count per total playing time | F |

**Supplementary figure 1-A.** The visualized description of touch data driven features used in the main input.


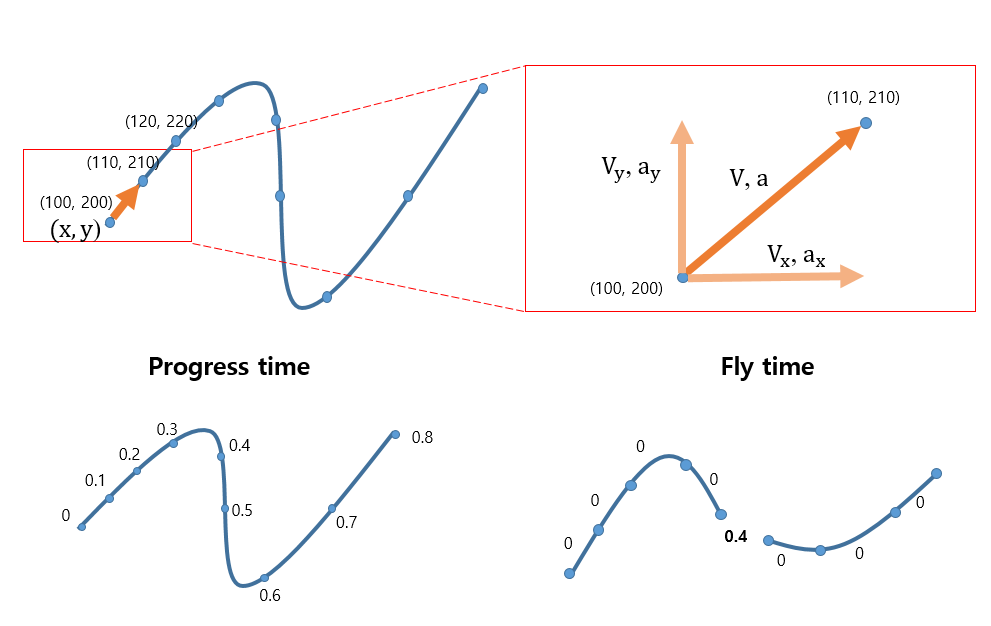


**Supplementary figure 1-B.** The visualized description of touch data driven features used in the main input.


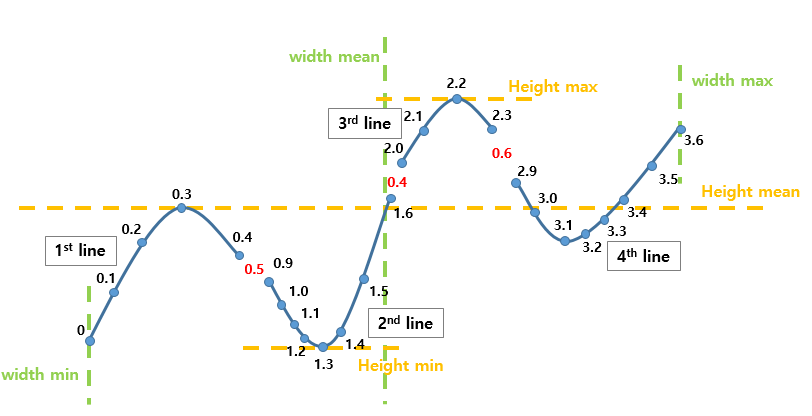

Supplement: Multimedia Appendix 2 [file games_v9i2e23130_app2.docx]
