# Supplementary material for: A Prediction Model for Detecting Developmental Disabilities in Preschool-Age Children Through Digital Biomarker-Driven Deep Learning in Serious Games: Development Study
Source: JMIR Serious Games. 2021 Jun 4;9(2):e23130. doi: 10.2196/23130 (PMC8214184; doi:10.2196/23130)
Supplement: Multimedia Appendix 3 [file games_v9i2e23130_app3.docx]

Multimedia appendix 3. Velocity and acceleration of movement (extended data of table 2)

| Characteristics | | Second game | | *P* value | CLES^a^ |
| --- | --- | --- | --- | --- | --- |
|  |  | Children with typical development | Children with developmental disabilities |  |  |
| Number of children | | 222 | 144 |  |  |
|  | **Velocity, median (IQR)** |  |  |  |  |
|  | $V_{x}$ sign (+/-), change count (n/game) | 1.0 (5.0) | 2.0 (4.0) | .40 | 0.429 |
|  | $V_{y}$ sign (+/-), change count (n/game) | 2.0 (6.0) | 2.0 (6.0) | .47 | 0.441 |
|  | $V_{x}$ sign (+/-), change count per line length (n/line) | 0.44 (1.23) | 0.53 (0.92) | .36 | 0.460 |
|  | $V_{y}$ sign (+/-), change count per line length (n/line) | 0.63 (1.51) | 0.66 (1.08) | .27 | 0.489 |
|  | **Accelerator, median (IQR)** |  |  |  |  |
|  | $A_{x}$ sign (+/-), change count (n/game) | 12.0 (20.25) | 10.0 (13.25) | .004 | 0.567 |
|  | $A_{y}$ sign (+/-), change count (n/ game) | 13.0 (22.25) | 11.0 (15.0) | .009 | 0.559 |
|  | $A_{y}$ sign (+/-), change count per line length (n/line) | 4.27 (4.47) | 3.05 (2.33) | <.001 | 0.628 |
|  | $A_{y}$ sign (+/-),change count per line length (n/line) | 4.27 (4.47) | 3.05 (2.34) | <.001 | 0.625 |
|  |  | Third game | |  |  |
| Number of children | | 216 | 145 |  |  |
|  | **Velocity, median (IQR)** |  |  |  |  |
|  | $V_{x}$ sign (+/-), change count (n/game) | 2.0 (6.0) | 2.0 (5.0) | .17 | 0.457 |
|  | $V_{y}$ sign (+/-), change count (n/game) | 4.0 (7.0) | 4.0 (7.0) | .03 | 0.524 |
|  | $V_{x}$ sign (+/-), change count per line length (n/line) | 0.4 (1.08) | 0.5 (0.87) | .36 | 0.461 |
|  | $V_{y}$ sign (+/-), change count per line length (n/line) | 0.89 (1.45) | 1.0 (0.96) | .11 | 0.534 |
|  | **Accelerator, median (IQR)** |  |  |  |  |
|  | $A_{x}$ sign (+/-), change count (n/game) | 15.0 (23.0) | 12.0 (21.0) | .01 | 0.555 |
|  | $A_{y}$ sign (+/-), change count (n/ game) | 16.0 (26.0) | 13.0 (18.0) | .04 | 0.543 |
|  | $A_{y}$ sign (+/-), change count per line length (n/line) | 4.33 (4.62) | 3.02 (2.73) | <.001 | 0.628 |
|  | $A_{y}$ sign (+/-),change count per line length (n/line) | 4.33 (4.61) | 3.02 (2.79) | <.001 | 0.627 |
|  |  | Sixth game | |  |  |
| Number of children | | 198 | 139 |  |  |
|  | **Velocity, median (IQR)** |  |  |  |  |
|  | $V_{x}$ sign (+/-), change count (n/game) | 4.0 (11.0) | 3.5 (7.0) | .08 | 0.506 |
|  | $V_{y}$ sign (+/-), change count (n/game) | 6.0 (14.5) | 6.0 (10.0) | .40 | 0.484 |
|  | $V_{x}$ sign (+/-), change count per line length (n/line) | 0.81 (1.26) | 0.66 (0.86) | .01 | 0.553 |
|  | $V_{y}$ sign (+/-), change count per line length (n/line) | 1.14 (1.98) | 1.13 (1.15) | .43 | 0.502 |
|  | **Accelerator, median (IQR)** |  |  |  |  |
|  | $A_{x}$ sign (+/-), change count (n/game) | 31.0 (51.5) | 15.0 (29.25) | <.001 | 0.635 |
|  | $A_{y}$ sign (+/-), change count (n/ game) | 34.0 (59.5) | 17.5 (29.5) | <.001 | 0.626 |
|  | $A_{y}$ sign (+/-), change count per line length (n/line) | 6.01 (4.89) | 3.42 (3.43) | <.001 | 0.697 |
|  | $A_{y}$ sign (+/-),change count per line length (n/line) | 6.01 (4.89) | 3.86 (3.21) | <.001 | 0.667 |

^a^CLES: common language effect size
