# Supplementary material for: A Prediction Model for Detecting Developmental Disabilities in Preschool-Age Children Through Digital Biomarker-Driven Deep Learning in Serious Games: Development Study
Source: JMIR Serious Games. 2021 Jun 4;9(2):e23130. doi: 10.2196/23130 (PMC8214184; doi:10.2196/23130)
Supplement: Multimedia Appendix 4 [file games_v9i2e23130_app4.docx]

Multimedia appendix 4. Hyperparameter optimization

For 1-D CNN, we conducted hyper-parameter optimization : for activation function {relu, elu, tanh, selu} for regularization strategy, within {L1($\left\| w \right\|_{1})$, L2($\left\| w \right\|_{2})$, L1+L2($\left\| w \right\|_{2}$)} and penalty value ($\lambda)$ within {0.01, 0.001, 0.0001, 0.00001} in each convolutional layer. Optimizer were searched within {RMSprop, Adamax}, and learning rate were done with {0.001, 0.005, 0.009}, epochs also were done {100, 150, 200, 250, 300, 350, 400}. Finally, we choose hyper-parameters as follow:

{optimizer: Adam, Regularization scheme: L2($\left\| w \right\|_{2})$, Regularization penalty value($\lambda)$: 0.00001, epoch: 350, activation function in all convolutional layers: selu}
